# Adaptation to cell culture induces functional differences in measles virus proteins

**DOI:** 10.1186/1743-422X-5-129

**Published:** 2008-10-27

**Authors:** Bettina Bankamp, Judith M Fontana, William J Bellini, Paul A Rota

**Affiliations:** 1 Measles, Mumps, Rubella and Herpesvirus Laboratory Branch, Division of Viral Diseases, Centers for Disease Control and Prevention, MS C-22, 1600 Clifton Road, Atlanta, Georgia 30333, USA; 2 Department of Microbiology and Immunology, Uniformed Services University of the Health Sciences, 4301 Jones Bridge Road, Bethesda, Maryland 20814, USA

## Abstract

**Background:**

Live, attenuated measles virus (MeV) vaccine strains were generated by adaptation to cell culture. The genetic basis for the attenuation of the vaccine strains is unknown. We previously reported that adaptation of a pathogenic, wild-type MeV to Vero cells or primary chicken embryo fibroblasts (CEFs) resulted in a loss of pathogenicity in rhesus macaques. The CEF-adapted virus (D-CEF) contained single amino acid changes in the C and matrix (M) proteins and two substitutions in the shared amino terminal domain of the phosphoprotein (P) and V protein. The Vero-adapted virus (D-VI) had a mutation in the cytoplasmic tail of the hemagglutinin (H) protein.

**Results:**

In vitro assays were used to test the functions of the wild-type and mutant proteins. The substitution in the C protein of D-CEF decreased its ability to inhibit mini-genome replication, while the wild-type and mutant M proteins inhibited replication to the same extent. The substitution in the cytoplasmic tail of the D-VI H protein resulted in reduced fusion in a quantitative fusion assay. Co-expression of M proteins with wild-type fusion and H proteins decreased fusion activity, but the mutation in the M protein of D-CEF did not affect this function. Both mutations in the P and V proteins of D-CEF reduced the ability of these proteins to inhibit type I and II interferon signaling.

**Conclusion:**

Adaptation of a wild-type MeV to cell culture selected for genetic changes that caused measurable functional differences in viral proteins.

## Background

The live attenuated vaccines currently used to protect against infection by measles virus (MeV) were developed well in advance of modern molecular biologic techniques, and the genetic basis of the attenuation of these vaccine strains remains a subject of investigation. The vaccines were generated by extensive passaging in cell culture, often involving cells or tissues of avian origin [1-4]. The identification of genomic markers for the attenuation of MeV would facilitate surveillance of wild-type MeVs by providing a means to discriminate between wild-type viruses and vaccine strains of the same genotype. In addition, this genetic information could be used to monitor the safety and stability of new vaccine lots and could contribute to the development of improved vaccines for MeV. The complete genomic sequences of many vaccine strains have been published [5-7]; however, the wild-type progenitors are no longer available for comparison or have undergone passaging in cell culture [8]. We have attempted to replicate the process of attenuation through cell culture adaptation with a wild-type MeV that is pathogenic for rhesus macaques [9,10].

MeV is a member of the genus *Morbillivirus *of the family *Paramyxoviridae*. Its monopartite, single-stranded, negative-sense RNA genome contains six genes, which encode eight proteins (reviewed in [11]). The non-coding regions of the termini contain the promoters for transcription and replication and the encapsidation signals. The nucleoprotein (N, 60 kDa) encapsidates the viral genome and binds the polymerase complex. The P gene encodes three proteins, the phosphoprotein (P, 72 kDa) and the C (21 kDa) and V (40 kDa) proteins. C is translated from an overlapping reading frame while V shares an amino terminal domain (NTD) of 231 amino acids with P, but has a unique carboxyl terminus as a result of RNA editing [12]. P is a necessary component of the polymerase complex and acts as a scaffolding protein in nucleocapsid assembly. It also contributes to the inhibition of type I interferon (IFN) signaling in infected cells [13]. The C and V proteins regulate polymerase activity [14-17] and act as inhibitors of IFN signaling [18-20]. The matrix protein (M, 38 kDa) plays a role in viral assembly and in the transport of viral glycoproteins to the apical membrane of polarized cells [21]. It also affects virus-induced fusion in cell culture [22,23]. The fusion (F) and hemagglutinin (H, 78 kDa) glycoproteins are expressed on the surface of infected cells and of the virion. The F protein is a disulfide-linked dimer (41 and 20 kDa) which promotes fusion with adjacent membranes. The H protein binds to specific receptors on the host cell and is a required co-factor for fusion [24]. The Large protein (L, 200 kDa) acts as the catalytic subunit of the polymerase complex.

In order to recreate the process of attenuation through cell culture adaptation, the D87-wt virus, which is pathogenic in rhesus macaques [10], was passaged in Vero cells, Vero/hSLAM cells and primary chicken embryo fibroblasts (CEFs) [9]. Vero cells (African green monkey kidney cells) express a homologue of CD46 which serves as a receptor for cell culture-adapted MeV strains [25-27]. Vero/hSLAM cells express both CD46 and human SLAM (signaling lymphocyte activation molecule), which is used as a receptor by all MeV strains [28-31]. CEFs do not express either of the two known receptors for MeV [32]. After nine passages in Vero/hSLAM cells, the resulting virus stock, D-V/S, remained genetically identical to D87-wt and retained pathogenicity in rhesus macaques. The Vero cell-adapted virus, D-VI, contained one amino acid substitution in the cytoplasmic tail of the H protein, while the CEF-adapted virus, D-CEF, contained four amino acid changes in the P, C, V and M proteins. Both viruses demonstrated attenuation in rhesus macaques. None of the viruses were able to infect Chinese hamster ovary cells expressing the receptor CD46, indicating that they had not adapted to use CD46 as a receptor [9]. The absence of a change in receptor usage indicated that, in this case, attenuation was a result of genetic changes affecting viral maturation, replication or interaction of viral proteins with intracellular host proteins.

In order to understand the consequences of the amino acid substitutions found in D-VI and D-CEF for protein functions, *in vitro *assays were used to analyze specific functions of the P, C, V, M and H proteins of the cell culture-adapted viruses. The effect of mutations in P, C, V and M on viral replication was examined with mini-genome replication assays. Quantitative fusion assays were used to analyze the role of the substitutions in M and H proteins in cell-cell fusion. The effect of the substitutions in P, C and V on IFN signaling was analyzed with reporter proteins expressed under the control of IFN-inducible promoters.

## Results

### Transient expression of wild-type and mutant proteins

**Figure 1:**
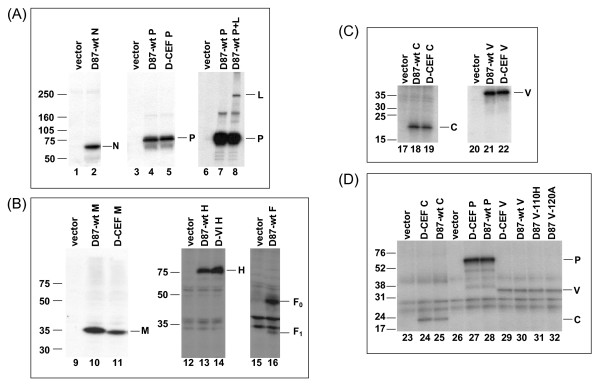
**Expression of proteins derived from D87-wt, D-VI and D-CEF**. (A, B, C) A549 cells were infected with vTF7-3 and transfected with the indicated pTM1-derived plasmids. (D) Vero cells were transfected with the indicated pCAGGS-derived plasmids. In all cases, proteins were labeled with ^35^S-methionine, precipitated with protein-specific antisera and separated by SDS-PAGE. Molecular mass markers (kDa) are shown on the left in each panel, the positions of proteins are indicated on the right.

Adaptation of D87-wt to CEFs resulted in the introduction of four amino acid changes, V102A in the C protein, Y110H and V120A in the NTD of the P and V proteins, and T84I in the M protein. Adaptation of D87-wt to Vero cells introduced one amino acid substitution in the H protein, L30P [9]. The ORFs for all eight proteins expressed by D87-wt as well as for the P, C, V and M proteins of D-CEF and the H protein of D-VI were cloned into the expression vector pTM1 behind a T7 promoter. The C ORF was silenced in the plasmids encoding P and V clones without affecting the amino acid sequence of the P and V proteins. Radio-immunoprecipitations of transiently expressed proteins demonstrated that all clones expressed proteins of the expected molecular sizes (figure 1A, B, C). The D87-wt L protein was co-expressed and co-immunoprecipitated with D87-wt P, using an antiserum to P (figure 1A, lanes 6–8). The M protein of D-CEF migrated at a slightly lower apparent molecular weight than the wt M protein (figure 1B, lanes 10, 11). Such differences in apparent molecular weight have been reported previously for M proteins of several MeV strains [33,34]. The P, C and V ORFs of D-CEF and the P ORF of D87-wt were subcloned into the mammalian expression vector pCAGGS to facilitate transcription by cellular RNA polymerases. Cloning of the D87-wt C and V ORFs into pCAGGS was described previously [35]. D87 V-110H and D87 V-120A each contained one of the two mutations identified in the V protein of D-CEF. Expression of P, C, and V proteins from pCAGGS was demonstrated by immunoprecipitation (figure 1D).

### Activity of P, C, V and M proteins in mini-genome replication assays

**Figure 2:**
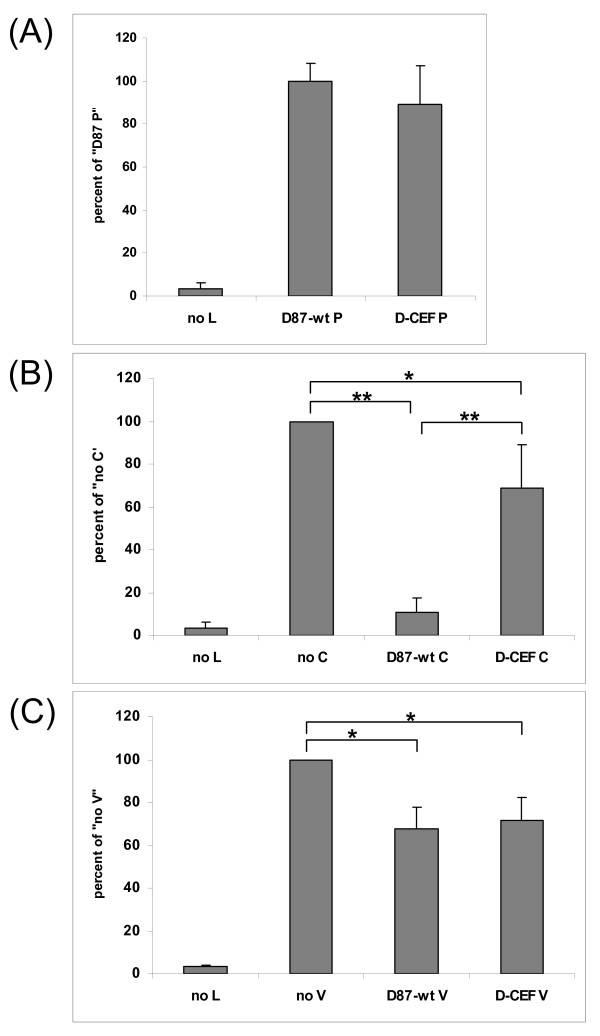
**Effect of mutations in the P, C and V proteins of MeV on mini-genome replication**. (A) CV-1 cells were infected with MVAT7 and transfected with pMV107(-)CAT, pTM1-D87-wt N, pTM1-D87-wt L and the indicated plasmids expressing P proteins. (B, C) CV-1 cells were infected with MVAT7 and transfected with pMV107(-)CAT, pTM1-D87-wt N, pTM1-D87-wt P, pTM1-D87-wt L and 1 μg of the indicated plasmids. For the negative controls, pTM1-D87-wt L was omitted. The amount of transfected DNA was kept constant through the addition of pTM1 vector. CAT protein production in cytoplasmic extracts of quadruplicate samples was measured by ELISA. The amount of CAT protein measured in the presence of pTM1-D87-wt P and absence of C- or V-expressing plasmids was set to 100% in each panel. Each panel shows the average of three independent experiments. Error bars denote one standard deviation. (*: P ≤ 0.01, **: P ≤ 0.001)

A mini-genome replication assay was used to analyze the ability of D87-wt P and D-CEF P to support polymerase activity. In this and all subsequent experiments, the N and L proteins were derived from D87-wt. As expected, both D87-wt P and D-CEF P supported replication equally well (figure 2A). In all subsequent replication assays, the D87-wt P protein was used.

The C and V proteins of different strains of MeV inhibit mini-genome replication to varying degrees [14,15,36]. The D87-wt C protein reduced CAT protein production by 89%, while the D-CEF C protein inhibited CAT production by only 31% (figure 2B). D87-wt V and D-CEF V reduced CAT protein production by 68% and 72%, respectively (figure 2C). These results demonstrate that the two amino acid substitutions in the NTDs of the P and V proteins of D-CEF did not affect the function of either protein in the mini-genome replication assay. In contrast, the single amino acid difference between the C proteins of the wild-type and the cell culture-adapted virus lead to a significant difference in their ability to inhibit replication.

**Figure 3:**
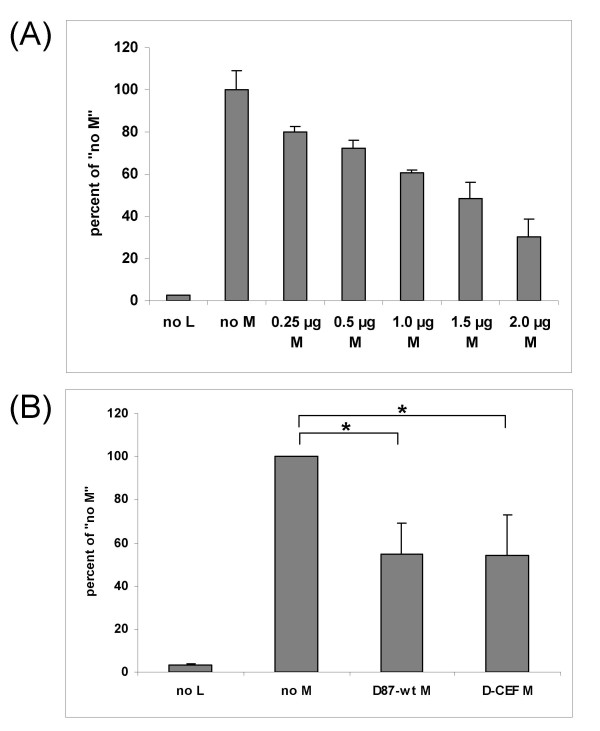
**The M protein of MeV inhibits mini-genome replication**. (A) CV-1 cells were infected with MVAT7 and transfected with pMV107(-)CAT, pTM1-D87-wt N, pTM1-D87-wt P, pTM1-D87-wt L and increasing amounts of pTM1-D87-wt M. (B) CV-1 cells were infected with MVAT7 and transfected with pMV107(-)CAT, pTM1-D87-wt N, pTM1-D87-wt P, pTM1-D87-wt L and 2 μg of the indicated plasmids. Transfections and ELISA were performed as described in the legend to figure 1. (*: P ≤ 0.01)

Previous reports showed that the M protein of MeV can inhibit polymerase activity [34,37]. We have confirmed that co-expression of the M protein in the mini-genome replication assay leads to a reduction in reporter protein levels in a dose-dependent manner (figure 3A). Figure 3B demonstrates that both D87-wt M and D-CEF M reduced CAT protein production by 45%, indicating that the single amino acid substitution in D-CEF M did not affect the level of inhibition.

### Activity of H and M proteins in quantitative fusion assays

**Figure 4:**
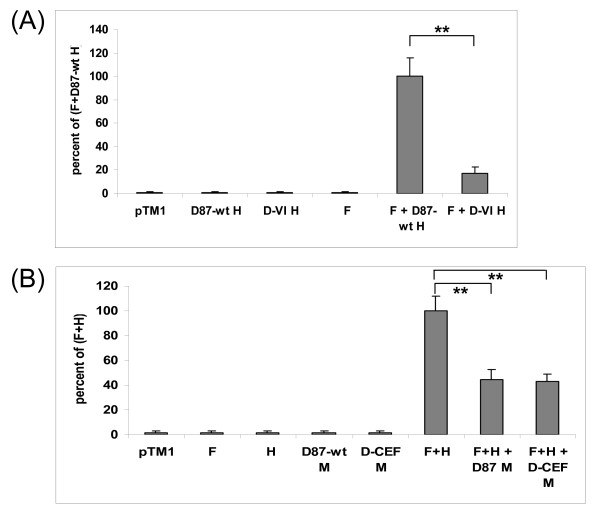
**Effect of mutations in the H and M proteins of MeV on quantitative fusion**. (A) fusion produced by H and F proteins, (B) inhibition of fusion by co-expressed M protein. Vero/hSLAM cells were infected with MVAT7 and transfected with the indicated plasmids. The amount of transfected DNA was kept constant through the addition of pTM1 vector. β-galactosidase protein production in cytoplasmic extracts of quadruplicate samples was measured as described in the Methods section. The amount of β-galactosidase protein measured in the wells transfected with pTM1-D87-wt F and pTM1-D87-wt H was set to 100% in each panel. Each panel shows the average of three independent experiments. Error bars denote one standard deviation. In panels (A) and (B) F indicates D87-wt F, in panel (B), H indicates D87-wt H. (**: P ≤ 0.001)

Mutations in the cytoplasmic tails of the F and H proteins can affect fusion [38-40]. A quantitative fusion assay with transiently expressed F and H proteins was used to measure the extent of fusion support provided by the H proteins of D87-wt and D-VI. Co-expression of D-VI H instead of D87-wt H resulted in an 82% reduction in reporter protein activity (figure 4A). Radioimmunoprecipitation experiments demonstrated that both H proteins were expressed equally well on the surface of transfected cells (data not shown).

Co-expression of M proteins with F and H proteins can modulate fusion activity, both in *in vitro *assays and in the intact virus [22,23]. Co-expression of wild-type or D-CEF M proteins reduced fusion significantly compared to the expression of F and H alone (figure 4B). The M proteins of D87-wt and D-CEF inhibited fusion by 56% and 57%, respectively, demonstrating that there was no difference in inhibition between the two M variants. These results showed that the amino acid substitution in M did not affect fusion inhibition, while a single amino acid substitution in the cytoplasmic tail of D-VI H had a significant effect on the H protein's ability to support fusion.

### Effect of P, C, and V proteins on IFN-β signaling

**Figure 5:**
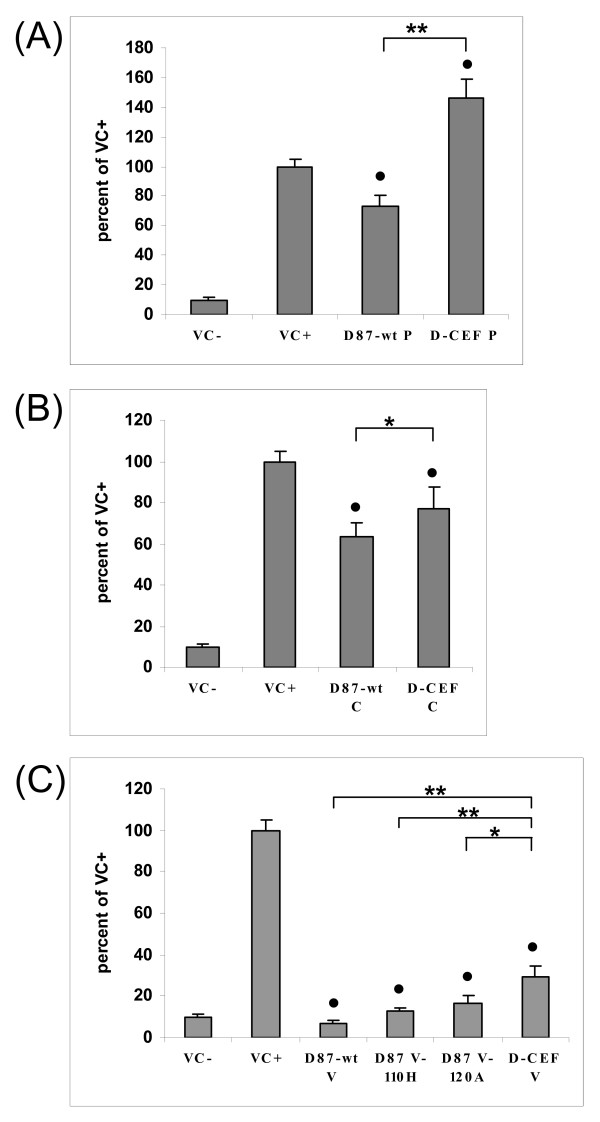
**Cell culture adaptation affects the inhibition of IFN-β signaling by the P, C, and V proteins of MeV**. Vero cells were transfected with a plasmid constitutively expressing renilla luciferase, a plasmid expressing firefly luciferase under the control of an IFN-α/β-responsive promoter and the plasmids as indicated in the three panels. Forty-eight hours after transfection, cells were stimulated with IFN-β for six hours, lysed and tested for luciferase activity. VC-indicates cells transfected with pCAGGS empty vector but not stimulated while VC+ indicates cells transfected with pCAGGS empty vector and stimulated with IFN-β. Results are expressed as a ratio of firefly to renilla luciferase luminescence taken as a percentage of the luminescence obtained using IFN-stimulated, empty pCAGGS vector (VC+). (A) results of IFN-β signaling assay with P proteins from D87-wt and D-CEF, (B, C) inhibition of IFN-β signaling by the C and V proteins, respectively. The data shown are an average of three experiments done with triplicate samples. Error bars denote one standard deviation. Bars marked with a are significantly different from VC+ with a P ≤ 0.05. (*: P ≤ 0.01, **: P ≤ 0.001)

The ability of D87-wt V and C to inhibit IFN signaling was described previously [35]. In this report, the P, C, and V proteins of D87-wt and D-CEF were compared in their ability to reduce the expression of an IFN-β-responsive reporter gene. D87-wt P inhibited luciferase expression by 27%, while D-CEF P lost the ability to inhibit IFN-β signaling (figure 5A). D87-wt C and D-CEF C reduced IFN-β signaling by 37% and 23%, respectively (figure 5B). D87-wt V and D-CEF V decreased reporter protein expression by 93% (14.7 fold) and 71% (3.4 fold), respectively (figure 5C). The V protein of D87-wt was modified to contain the amino acid substitution Y110H or V120A found in D-CEF V, and these mutant V proteins demonstrated intermediate levels of inhibition (figure 5C). Our results show that all three wild-type proteins inhibited IFN-β signaling more effectively than did the corresponding protein from D-CEF. The most potent inhibitor of signaling was the V protein, followed by C and P. Both mutations in the NTD of D-CEF V contributed to its reduced ability to inhibit IFN-β signaling, but even with both mutations, D-CEF V still retained significant inhibitory potential.

### Effect of P and V proteins on IFN-γ signaling

**Figure 6:**
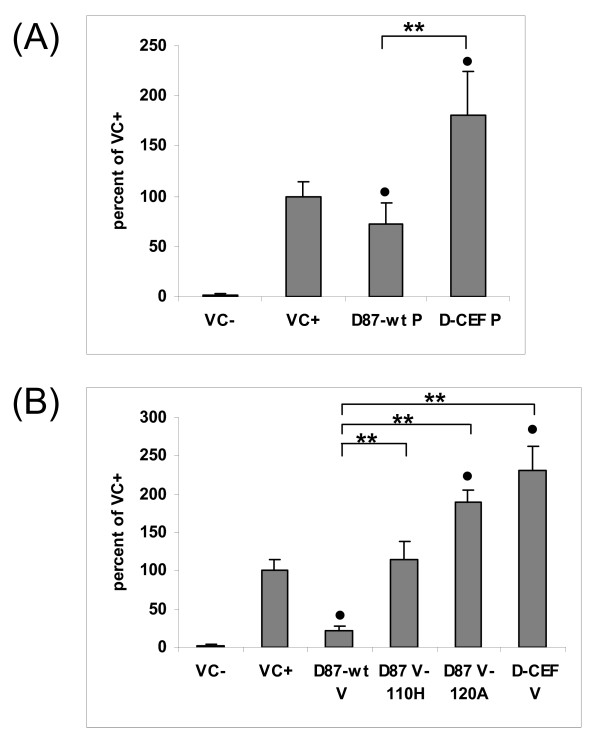
**Cell culture adaptation affects the inhibition of IFN-γ signaling by the P and V proteins of MeV**. Experiments were performed as described in the legend to figure 5, except that a reporter plasmid expressing firefly luciferase under the control of an IFN-γ-responsive promoter and IFN-γ were used for stimulation. (A) results of IFN-γ signaling assays with P proteins from D87-wt and D-CEF, (B) inhibition of IFN-γ signaling by the V proteins. Bars marked with a are significantly different from VC+ with a P ≤ 0.05. (**: P ≤ 0.001)

The P and V proteins of D87-wt and D-CEF were compared in their ability to inhibit the expression of an IFN-γ-responsive reporter gene. We reported previously that the C protein of MeV does not inhibit IFN-γ signaling [35]. As expected, neither the C protein of D87-wt nor that of D-CEF inhibited the expression of an IFN-γ-responsive reporter gene (data not shown). D87-wt P reduced luciferase expression by 28%, while D-CEF P did not inhibit IFN-γ signaling (figure 6A). D87-wt V reduced IFN-γ signaling by 78%, while D-CEF V lost the ability to inhibit reporter gene expression (figure 6B). Each of the two mutants containing one or the other of the amino acid substitutions of D-CEF V failed to inhibit IFN-γ signaling. These results showed that the P and V proteins of D87-wt inhibited IFN-γ signaling more effectively than did the corresponding proteins from D-CEF and that V was a more potent inhibitor than P. Each of the substitutions found in the V protein of D-CEF individually caused a complete loss of this activity.

## Discussion

The C protein of D-CEF demonstrated a significantly reduced ability to inhibit reporter protein expression in a mini-genome replication assay. We previously showed that naturally occurring substitutions between amino acids 45 and 167 of the MeV C protein modified its ability to regulate polymerase activity [14]. Therefore, the single amino acid substitution (V102A) of the D-CEF C protein lies within a domain of the C protein that regulates replication. Recombinant MeVs defective in expression of the C protein induce more IFN than wild-type viruses, indicating that the increased production of RNA may activate cellular RNA sensors [41]. However, it is still unclear which effect mutations in this domain have on attenuation. In addition to regulating polymerase activity, the C protein participates in the inhibition of the host IFN response and cell death and acts as an infectivity factor that improves particle release [19,35,42,43]. Since the MeV C protein is a multifunctional protein, it is difficult to separate the effects that amino acid substitutions have on each of its activities; however, *in vitro *assays are useful tools to measure individual functions.

The V protein of MeV has been shown to regulate replication both in mini-genome assays and in infected cells [16,36]. Mutational analysis characterized two domains involved in inhibition of mini-genome replication, amino acids 113 and 114 in the NTD and amino acids 238–278 in the unique carboxyl terminus [17,36]. Amino acids 110–131 are highly conserved among morbilliviruses [17]. Despite the proximity of the mutations found in D-CEF V (amino acids 110 and 120) to this conserved region of V and P, these substitutions had no effect on reporter protein production in the mini genome replication assay.

The M protein of MeV inhibited *in vitro *transcription of purified nucleocapsids, and M proteins of different MeVs varied in their ability to inhibit *in vitro *transcription [34]. SiRNAs directed against the M gene increased replication, transcription and protein expression of other structural proteins [37]. A dose-dependent inhibition of reporter protein production in the mini-genome replication assay confirmed the earlier findings that M inhibits transcription and/or replication. However, there was no difference in the level of inhibition between the wild-type and mutant M proteins. Since both M proteins inhibited fusion to the same extent, the role of the mutation in D-CEF M remains unknown. While three of the four amino acid substitutions in D-CEF resulted in functional differences in the *in vitro *assays, it will be necessary to create a recombinant virus containing only the mutation in M to measure its effect on viral replication.

The L30P substitution in the cytoplasmic tail of the D-VI H protein increased titers of extracellular virus in Vero cells [9]. In this report, we demonstrated that the substitution led to a significant reduction in fusion help in Vero/hSLAM cells. Since neither D-V/S nor D-VI induced fusion in Vero cells (data not shown), the alteration in fusion measured in Vero/hSLAM cells probably does not indicate that fusion capacity itself played a role in cell culture adaptation. However, alterations in the cytoplasmic tails of MeV glycoproteins can modulate the interaction of F and H proteins, which affects fusion [38-40]. We hypothesize that decreased fusion indicates a stronger interaction between F and H proteins, which may affect titers. An alternative interpretation of our data is based on the observation that expression of MeV glycoproteins and/or M leads to the formation of virus-like particles (VLPs) [44]. A decrease in fusion may be the result of increased budding, reducing the amount of available F and H on the surface of the effector cells. VLPs could also interact with the indicator cells in the fusion assay, acting as soluble inhibitors of fusion.

**Figure 7:**
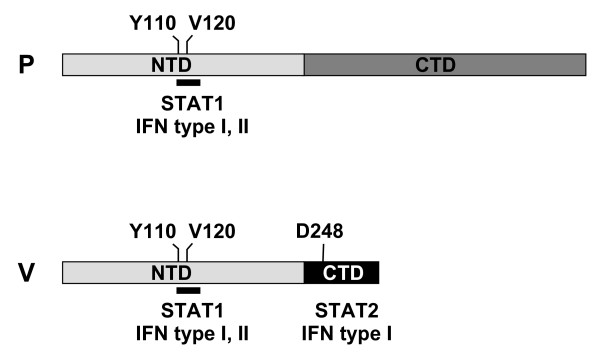
**Location of IFN-inhibiting domains in the P and V proteins of MeV**. P indicates P protein, V indicates V protein, NTD indicates shared amino terminal domain, and CTD indicates unique carboxyl terminal domain. The black bars denote the domain from amino acids 110–130 in the NTD. Positions of important amino acids are marked.

In this first report to directly compare the ability of the P, C, and V proteins of MeV to inhibit both IFN-β and IFN-γ signaling, the V protein was clearly the most potent inhibitor of both signaling pathways. Although the P protein of MeV has been previously shown to inhibit the expression of an IFN-α/β-responsive reporter gene [13], we did not find the P protein of D87-wt to be as strong an inhibitor of either IFN-β or IFN-γ signaling as the previous report indicated. This discrepancy may be due to the different cell types used in each study. Amino acid 110 in the NTD of the P and V proteins plays a critical role in the ability of MeV to inhibit IFN signaling [13,35,45], but this is the first report to demonstrate the contribution of amino acid residue 120 to this activity. Amino acids 110–131 are highly conserved among morbilliviruses [17] and bind STAT1 [46], which explains why the shared NTD inhibits both type I and type II IFN signaling. The unique carboxyl terminal domain of V binds STAT2 [46], suggesting that it can only inhibit type I IFN signaling. Our findings, combined with those of previous publications [13,45-47], are summarized in a model of the STAT-binding sites in the P and V proteins and the resulting IFN-signaling inhibition (figure 7).

Expression of P or V mutants that did not inhibit IFN signaling lead to reproducible apparent augmentation of reporter protein expression above the level of the positive control (figures 5A, 6). The mechanism behind this phenomenon is unclear. Yokota *et al*. [20] reported that the inducibility of an IFN-γ-responsive reporter gene was enhanced in MeV-infected CaSki (epitheloid carcinoma) cells compared to uninfected cells. Furthermore, treatment with IFN-γ caused a prolonged and enhanced phosphorylation of Jak1 and Jak2 in these MeV-infected cells [20]. The authors hypothesized that the enhanced phosphorylation may be the reason for increased reporter gene expression. It is unknown whether a similar enhanced phosphorylation of Jak proteins may also occur in plasmid-transfected cells expressing MeV proteins. Interaction of P or V with other cellular proteins, perhaps other IFN-inducible genes, may contribute to this effect.

We are faced with the paradox that adaptation to a presumably IFN-competent primary cell line such as CEFs induced a loss of the ability to counteract IFN signaling. Our hypothesis is that CEF adaptation induces substitutions that improve the interaction of viral proteins with avian proteins, perhaps even proteins involved in avian IFN signaling. IFN signaling inhibition by paramyxoviruses can be species specific, for example PIV5 cannot inhibit IFN-α/β signaling in murine cell lines [48]. Similar species specific adaptation may be a cause for the attenuation of MeV vaccine strains, many of which have been passaged extensively in avian cells or tissues [8]. Since the reduced ability to inhibit IFN signaling would presumably not affect the replication of D-CEF in Vero cells, the observed improvement in viral titers compared to D-V/S may be the result of the mutations in the C and M proteins. The construction of recombinant viruses expressing individual mutations identified in D-CEF will make it possible to examine the role of each substitution separately.

A number of studies have identified amino acid substitutions in most proteins of MeV [9,49,50] as a result of cell culture adaptation. A comparison of the sequences of five vaccine strains derived from the Edmonston progenitor with the Edmonston wt strain identified amino acid differences in every protein [6]. Replacement of ORFs in a recombinant wild-type MeV with ORFs from an attenuated virus demonstrated that multiple proteins contributed to cell culture adaptation [51,52]. It is likely that there are several pathways to attenuation and amino acid substitutions in multiple proteins may have a cumulative effect on pathogenicity.

## Conclusion

Adaptation of a wild-type MeV to Vero cells and CEFs selected for genetic changes that caused measurable functional differences in viral proteins. Our results demonstrate the usefulness of *in vitro *assays to characterize the consequences of cell culture adaptation. Identification of mutations that are associated with the alteration of protein function will increase our understanding of the pathogenicity of MeV. This knowledge can be used towards engineering recombinant strains of MeV that can be used therapeutically, such as oncolytic viruses, as well as towards the development of improved MeV vaccines.

## Methods

### Cells and viruses

A549, Vero, CV-1 and Vero/hSLAM cells [30] were maintained in Dulbecco's modified Eagle's medium supplemented with 10% fetal calf serum, glutamine and antibiotics. G418 sulfate (Cellgro) was used to maintain expression of hSLAM in Vero/hSLAM cells (0.4 mg ml^-1^). The MVAT7 and vTF7-3 recombinant vaccinia viruses were provided by B. Moss, Bethesda, MD, USA and were propagated in CEFs and Vero cells, respectively.

### Derivation of plasmids

The cloning of the C and V open reading frames (ORFs) of D87-wt has been described [35]. The same RNA preparations that were used for sequence analysis of D87-wt, D-CEF and D-VI [9] were used to derive cDNA for the N, P, M, F, H and L ORFs of D87-wt, the P, C, V and M ORFs of D-CEF and the H ORF of D-VI. Reverse transcriptase reactions were performed with an oligo (dT) primer and Superscript II reverse transcriptase (Invitrogen). PCR was performed with the Elongase enzyme mix (Invitrogen) using gene-specific primers. Primer sequences are available upon request. The start codon of the C protein was mutated in the P/V forward primer (ATG changed to ACG); the substitution is silent in P and V. The L gene was amplified in two fragments that overlapped at a unique NheI site (nt 12216). The genes were cloned into the expression vector pTM1 by using the following restriction sites: SacI and SpeI (N), EcoRI and SpeI (P, C, V), SpeI and PstI (M), SacI and XhoI (F), BamHI and EcoRI (H), BamHI and SalI (L). The mammalian expression vector, pCAGGS [53] was provided by C. Basler, Mount Sinai School of Medicine, New York, NY, USA. Restriction sites EcoRI and XhoI were used to subclone the P, C and V ORFs into pCAGGS, to which a linker containing *EcoRI *and *XhoI *recognition sites had been added. D87-V-120A and D87-V-110H were generated from D-CEF V by using a three-step PCR method with the P gene-specific primers and primers containing the desired mutation (underlined), V110F (5'-GCA CTG GGC TAC AGT GCT ATC ATG TTT ATG ATC ACA GCG G-3'), V110R (5'-CCG CTG TGA TCA TAA ACA TGA TAG CAC TGT AGC CCA GTG C-3'), V120F (5'-GCG GTG AAG CGG TTA AGG GAA TCC AAG-3'), V120R (5'-CTT GGA TTC CCT TAA CCG CTT CAC CGC-3'). The mutated amplicons were cloned into the pCAGGS vector using *EcoRI *and *XhoI*. All clones were sequenced using the ABI PRISM Dye Terminator Reaction Kit and the ABI 3100 and 3130xL Genetic Analyzer machines (Perkin Elmer-Applied Biosystems). Sequence data were analyzed with the Sequencher™ DNA sequencing program (Gene Codes Corporation) and confirmed by comparison to the published sequence for each strain. The mini-genome construct, pMV107(-)CAT [54], was a gift of M. Billeter (Zürich, Switzerland). pG1NT7, which carries the lac Z gene under control of the T7 promoter, was provided by B. Fredericksen, University of Maryland, MD, USA. The pISRE and pGAS plasmids were provided by R.E. Randall, North Haugh University of St. Andrews, Fife, Scotland. The pRL-TK plasmid was part of the Dual Reporter Luciferase System (Promega).

### Protein expression

For expression of genes cloned into pTM1, A549 cells in 6-well plates were infected with vTF7-3 at a multiplicity of infection (MOI) of 5 and transfected 45 min later. 2 μg plasmid DNA in Opti-MEM medium (Invitrogen) were transfected with Cellfectin (Invitrogen). pTM1 without a coding sequence was used as a negative control. Cells were starved for one hour in methionine-free medium (ICN), followed by labeling with ^35^S-methionine for one hour (N, P, C, V proteins) or four hours (L protein). For expression of M, F and H proteins, cells were labeled overnight in the presence of 1% FBS. Cytoplasmic cell extracts were prepared in NET-BSA buffer (150 mM NaCl, 5 mM EDTA, 50 mM Tris-HCl, 0.5% NP-40, 1 mg ml^-1 ^BSA, pH 7.4). Aliquots of cell extracts were incubated with protein-specific antisera (N, P, C, V, F, H) or monoclonal antibodies (M) followed by precipitation with GammaBind G-Sepharose (Amersham). The L protein was co-expressed with the P protein and co-precipitated with P specific antiserum. The complexes were separated on a 4–20% gradient SDS-polyacrylamide gel (Bio-Rad Laboratories) (N, P, C, V, L proteins) or a 10% SDS-polyacrylamide gel (Bio-Rad Laboratories) (M, F, H proteins). Bands were visualized by autoradiography. Expression of genes cloned into pCAGGS was performed as described previously, using the same antisera as described above [35].

### Replication assay

Mini-genome replication assays were performed as described previously [14]. In experiments including pTM1-C, -V or -M plasmids, transfected amounts are listed in the figures. CAT protein concentrations measured in cell extracts of cells transfected with wild-type N, P, L plasmids were set to 100%.

### Fusion assay

Vero/hSLAM cells in 12-well plates (effector cells) were infected with MVAT7 at an MOI of 5 and transfected with 10 ng pTM1-D87 F, 20 ng plasmid expressing H and/or 10 ng plasmid expressing M in Opti-MEM (Invitrogen) using Cellfectin (Invitrogen). pTM1 vector alone, or H, F or M expressing plasmids alone were transfected as negative controls. The amount of transfected DNA was kept constant through the addition of pTM1 vector. For every protein tested, two plasmid preparations were used, and for every sample, transfection mixtures were tested in duplicate. A second set of Vero/hSLAM cells in 6-well plates (indicator cells) was transfected with 3 μg pG1NT7 using TransIT-LT1 (Mirus Bio). 18 hours after transfection, indicator cells were detached with trypsin (Invitrogen) and added to the wells containing the effector cells. 4 to 5 hours later, expression of β-galactosidase in cytoplasmic extracts was measured with a luminescent substrate (Clontech), following the manufacturer's Alternate Cell Lysis Protocol. A Fluoroscan Ascent microplate luminometer (Thermo Electron) was used to measure luminescence. Purified β-galactosidase (Clontech) was used to establish a standard curve. β-galactosidase concentrations measured in lysates of cells transfected with wild-type F and H plasmids were set to 100%.

### IFN-responsive reporter gene assay

Experiments were carried out as described previously [35]. Briefly, Vero cells in 24-well plates were transfected with Opti-MEM medium (Invitrogen) and TransIT-LT1 (Mirus Bio). 0.9 μg pISRE and 0.1 μg pRL-tk or 0.7 μg pGAS and 0.3 μg pRL-tk were co-transfected with 1 μg of pCAGGS-P, -C, or -V plasmid DNA. 48 hours after transfection, cells were treated with 100 U rhIFN-β (Biosource International) for six hours and cell lysates were harvested for detection of luciferase activity using the Dual Reporter Luciferase System (Promega). Luminescence obtained using IFN-stimulated, pCAGGS-transfected cells was set to 100%.

### Statistical analysis

For two group comparisons, a two-tailed Student's *t*-test was used, and a value of p < 0.05 was considered statistically significant.

## Competing interests

The authors declare that they have no competing interests.

## Authors' contributions

BB participated in the design of the study, carried out the majority of the experiments and drafted the manuscript. JMF constructed pCAGGS-based expression plasmids, carried out the IFN-responsive reporter gene assays and helped to draft the manuscript. WJB revised the manuscript. PAR participated in the design of the study and revision of the manuscript. All authors read and approved the final manuscript.
